# Non-Chlamydial Bacterial Infection and Progression of Conjunctival Scarring in Trachoma

**DOI:** 10.1167/iovs.17-23381

**Published:** 2018-05

**Authors:** Victor H. Hu, David Macleod, Patrick Massae, Isaac Afwamba, Helen A. Weiss, David C. W. Mabey, Robin L. Bailey, Matthew J. Burton

**Affiliations:** 1 London School of Hygiene and Tropical Medicine, London, United Kingdom; 2 Kilimanjaro Christian Medical Centre, Moshi, Tanzania

**Keywords:** trachoma, scarring, bacteriology, cohort

## Abstract

**Purpose:**

The purpose of this study was to assess whether non-chlamydial bacterial infection is associated with progression of trachomatous scarring in adults.

**Methods:**

This was a cohort study involving 800 participants in northern Tanzania who underwent clinical examination, photography, and conjunctival swab collection for microbiology over a 24-month period. Samples for microbiology were inoculated onto blood and chocolate agar, and *Chlamydia trachomatis* was detected by PCR. Progression was determined by comparison of baseline to 24-month photographs.

**Results:**

*C. trachomatis* was detected in only four participants at baseline. At 24 months, 617 participants (77.1%) were followed up. Of those seen at 24 months, 452 could be reliably assessed. Definite scarring progression (progressors) was seen in 345 (55.9%); there was no progression (nonprogressors) in 107 (17.3%). Using combined baseline and 12-month microbiology results, progressors had significantly higher levels of commensal and pathogenic bacterial organisms detected compared with nonprogressors. After adjusting for age, baseline scarring, and ethnicity, there was weak evidence (*P* = 0.07) that the bacteria category was associated with scarring progression (commensal organisms only: odds ratio [OR] = 1.61; 95% confidence interval [CI]: 0.90 to 2.89; pathogenic organisms either with or without commensal: OR = 2.39; 95% CI: 1.10 to 5.16).

**Conclusion:**

The findings were consistent with the possibility that trachomatous scarring in adults is associated with the presence of non-chlamydial bacterial organisms, particularly pathogenic organisms. *C. trachomatis* was detected very infrequently and may not be an important factor in the pathogenesis of scarring progression in adults. This has implications for trachoma control programs, which largely concentrate on reducing *C. trachomatis* levels and transmission.

Trachoma is caused by recurrent episodes of infection with *Chlamydia trachomatis*. In childhood, infection is characterized by a follicular-papillary conjunctivitis.^1^ Such individuals are at risk of developing the scarring complications of trachoma in later life, including conjunctival scarring, entropion (the eyelid turning in toward the globe), and trichiasis (eyelashes rubbing against the globe). The result is corneal scarring and loss of vision. The development of scarring is a cumulative process. Therefore, the prevalence and severity of scarring increases with age. Trachoma control efforts are primarily focused on reducing *C. trachomatis* prevalence and transmission with the use of mass antibiotic administration and the improvement of hygiene and sanitation.

Infection with *C. trachomatis* shows a consistent relationship with active trachoma in children, especially in treatment-naïve populations.^2^ In contrast, the pathogenesis of progressive scarring in adults is less clear. *C. trachomatis* is only infrequently detected in ocular surface samples from adults who have trachomatous scarring, with detection rates sometimes being close to zero.^3–7^ A recent review on trachoma progression found only one study providing evidence that children with baseline *C. trachomatis* infection were at an increased risk of subsequent scarring and no studies demonstrating that progression of established scarring was associated with *C. trachomatis*.^7,8^ Despite this, adults with established scarring show progression of that scarring with the development of sight-threatening complications. Incident trichiasis has been observed in The Gambia after *C. trachomatis* had been effectively eliminated.^9^ Recent cohort studies from Ethiopia and Tanzania in adults found scarring progression in around one quarter of subjects after 2 years, despite negligible *C. trachomatis* DNA detection.^6^ This raises the question about whether there might be other factors that can contribute to progressive scarring processes.

A strong association has been found in longitudinal studies between conjunctival inflammation and progressive scarring, indicating a central role in the pathogenesis of cicatricial trachoma. The Ethiopian and Tanzanian scarring trachoma cohort studies reported an odds ratio (OR) of 5.8 (*P* < 0.0001) for progressive scarring with increasing episodes of clinical inflammation. Although the pathogenesis of scarring trachoma in adults is relatively poorly understood, recent studies have suggested that innate immune mechanisms driven by epithelial responses may be important.^1,10–12^ Non-chlamydial bacterial infection of the conjunctiva has been found to be associated with the presence of clinically visible inflammation, with trachomatous scarring (without trichiasis), with trachomatous trichiasis, and with recurrence after trichiasis surgery.^3,9,13–16^ This non-chlamydial bacterial infection is associated with the elevated expression of a variety of proinflammatory mediators and modifiers of the extracellular matrix.^10^ Inoculation of bacteria into eyes with scarring in a monkey model of trachoma caused a more marked and prolonged inflammatory reaction compared with control animals.^17^ In this study, we examined whether non-chlamydial bacteria cultured from the conjunctiva were associated with progression of trachomatous scarring over time.

## Methods

### Ethical Approval

This study adhered to the tenets of the Declaration of Helsinki. It was approved by the Ethics Committees of the Tanzanian National Institute for Medical Research, the Kilimanjaro Christian Medical Centre, and the London School of Hygiene & Tropical Medicine. The study was explained to potential study subjects, and written information in Kiswahili was provided. Informed consent was obtained before enrollment. If the participant was unable to write, consent was recorded by witnessed thumbprint, as approved by the Ethics Committees.

### Participant Recruitment and Examination

Recruitment of participants and clinical examination procedures have been previously described.^6^ Briefly, 800 adults with trachomatous upper tarsal conjunctival scarring, but without trichiasis, from a trachoma endemic region in Siha District, Northern Tanzania, were recruited into a 2-year longitudinal cohort study. Study participants were assessed at baseline and 6, 12, 18, and 24 months, including clinical examination and high-resolution digital photography of the everted upper tarsal conjunctiva of the left eye (Nikon D200 camera with Nikon 105-mm macro lens and Nikon R1 flash system; Nikon, Tokyo, Japan). Grading was performed using the detailed “FPC” World Health Organization Trachoma Grading System, with a more detailed scarring grading system as previously described.^13,18,19^ The left conjunctiva was anesthetized with preservative-free proxymetacaine 0.5% eye drops (Minims; Chauvin Pharmaceuticals, London, UK). A Rayon-tipped swab sample was collected for microbiological analysis from the inferior conjunctival fornix, immediately placed into Amies-Charcaol transport media (Sterilin, Caerphilly, UK), and kept at ambient temperature. An upper tarsal conjunctival swab was also collected (Dacron polyester-tipped; Hardwood Products Company, Guildford, ME, USA) and put into a dry tube for *C. trachomatis* detection. These samples were kept on ice packs until frozen later the same day at −80°C.

### Scarring Progression

To determine whether progression in conjunctival scarring had occurred, the baseline and 24-month tarsal conjunctival photographs were directly compared side by side by two ophthalmologists working independently. Individuals with progressive scarring, progressors, were defined as those with clear photographic evidence of increased conjunctival scarring at 24 months. Nonprogressors clearly did not have photographic evidence of scarring progression. Any disparities in progression status were discussed and agreement reached. Grading was performed with masking to all laboratory results.

### Microbiology Samples and Analysis

*C. trachomatis* DNA was detected using a PCR-based assay (Amplicor CT/NG Test; Roche Diagnostics, Indianapolis, IN, USA) with previously described modifications.^20^ Samples for culture were inoculated onto blood and chocolate agar later on the day of collection (rarely more than 6 hours) and incubated at 37°C for 48 hours. Culture isolates were identified by standard microbiological techniques. In keeping with previous work, coagulase-negative staphylococci, *Corynebacterium* spp., *Streptococcus viridans*, and *Bacillus* spp. were designated as commensal organisms.^13^ Other organisms were categorized as pathogenic at this site.

### Sample Size and Data Analysis

This study was part of a larger series of related studies on the pathogenesis of trachomatous scarring with the sample size calculated to encompass these other components. Data were entered into Access 2007 (Microsoft, Redmond, WA, USA) and analysed using STATA 14.0 (StataCorp LP, College Station, TX, USA). Fisher's exact test was used to determine strength of association for demographic and baseline characteristics with scarring progression. Culture results at baseline and 12 months were combined to form a single parameter with three categories. Individuals with a pathogenic organism at either time point were classified as the pathogenic group; individuals with a commensal but without a pathogenic organism at either time point were classified as the commensal group. If data were missing at one of the time points, the individual was classified using the data from the available single time point. If data were missing at both time points, the observation was classified as missing. Logistic regression models were used to estimate univariable ORs and 95% confidence intervals (CIs) for factors associated with scarring progression from baseline to 24 months. A multivariable logistic regression model was generated including factors independently associated with scarring progression, using a threshold of *P* < 0.1 as the criteria for inclusion. Likelihood ratio tests were used to assess the strength of association of each factor with the outcome and tests for nonlinearity were conducted to assess whether fitting age category and scarring as continuous variables provided adequate fits to the data.

## Results

Initially, 800 individuals with conjunctival scarring were recruited. Only four of these individuals were positive for *C. trachomatis* by PCR at baseline. At the 24-month follow-up, 617 (77.1%) of the study participants were seen and had photographs taken (the remainder had either died [18], moved away [72], or were absent/refused examination at follow-up [93]), with a mean follow-up time of 681 days (SD, 39 days). There were 452 of 617 (73.3%) individuals who had high-quality photographs at both baseline and 24 months, in whom the masked graders were confident that there was either progression in scarring, or no scarring progression, by 24 months. Baseline demographic details of those included and excluded from subsequent analyses are shown in Table 1; there was no significant differences between the two groups. There were 345 progressors and 107 nonprogressors. Progressors tended to be older than nonprogressors, had less education, and had more severe grades of conjunctival scarring at baseline (Table 2).

**Table 1:**
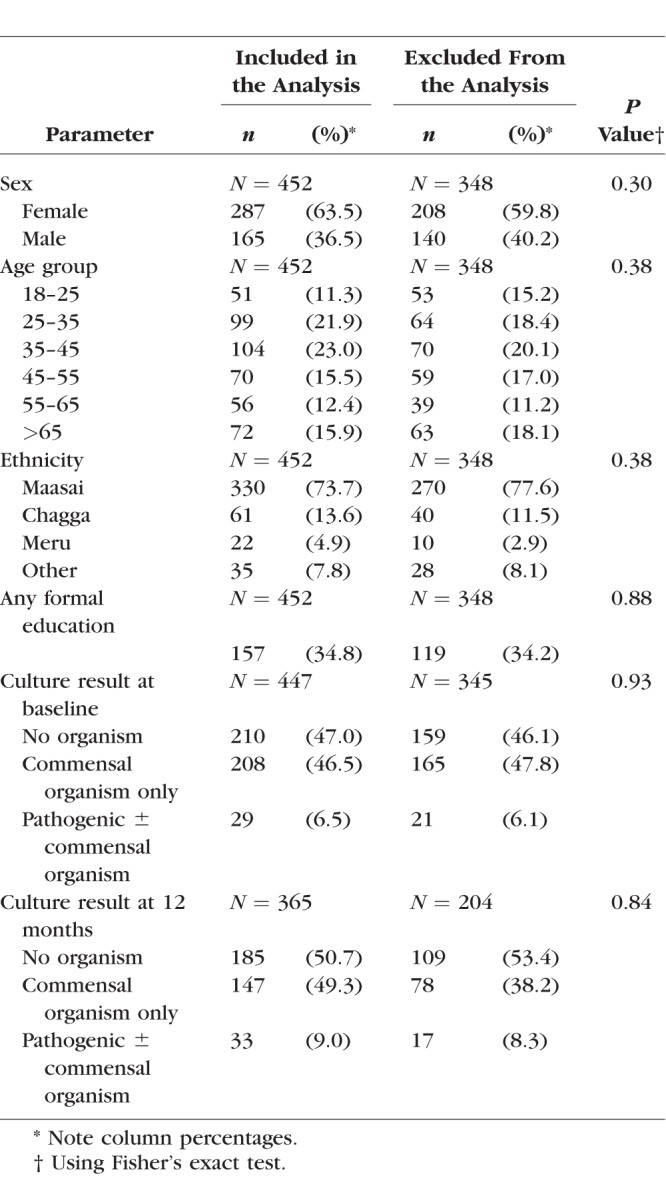
Demographic Characteristics of the Cohort Study Participants, Subdivided Into Those Included and Excluded From Progression Analysis

**Table 2:**
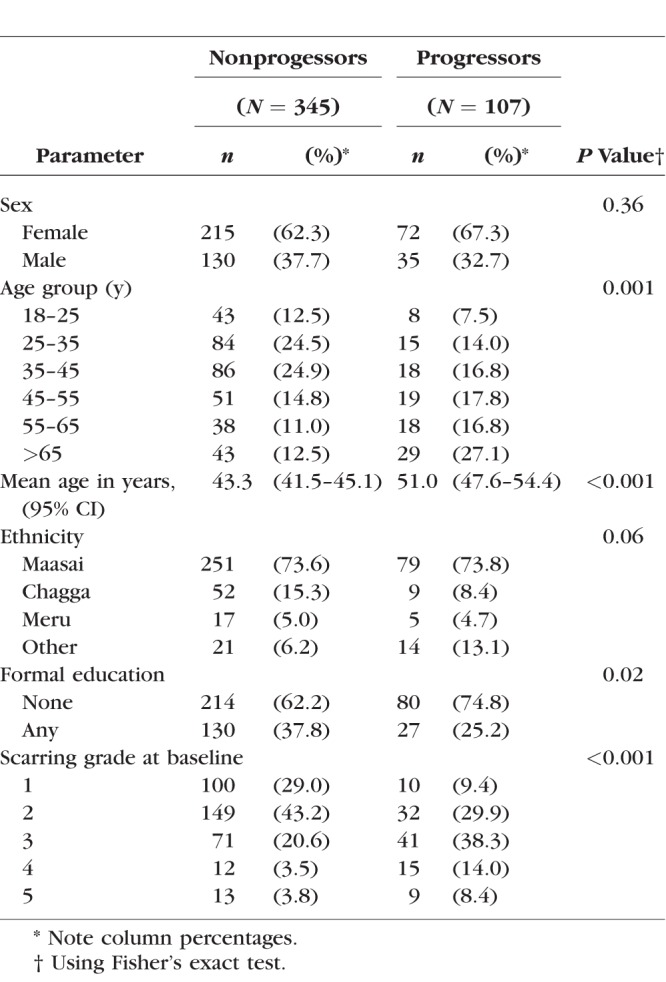
Demographic Characteristics and Baseline Scarring Grade, Subdivided Into Nonprogressors and Progressors

Microbiological culture results at baseline and 12 months, stratified by scarring progression status, are shown in Table 3. Progressors had a higher prevalence of both commensal and pathogenic organisms at both baseline and 12 months. The remaining results presented use the combined baseline/12-month results. Table 4 shows univariable ORs and a multivariable logistic regression model for factors associated with scarring progression by 24 months. After adjusting for age, baseline scarring, and ethnicity, there was some evidence that the bacteria category was associated with scarring progression (commensal organisms only: OR = 1.61; 95% CI: 0.90 to 2.89; pathogenic organisms either with or without commensal: OR = 2.39; 95% CI: 1.10 to 5.16).

**Table 3:**
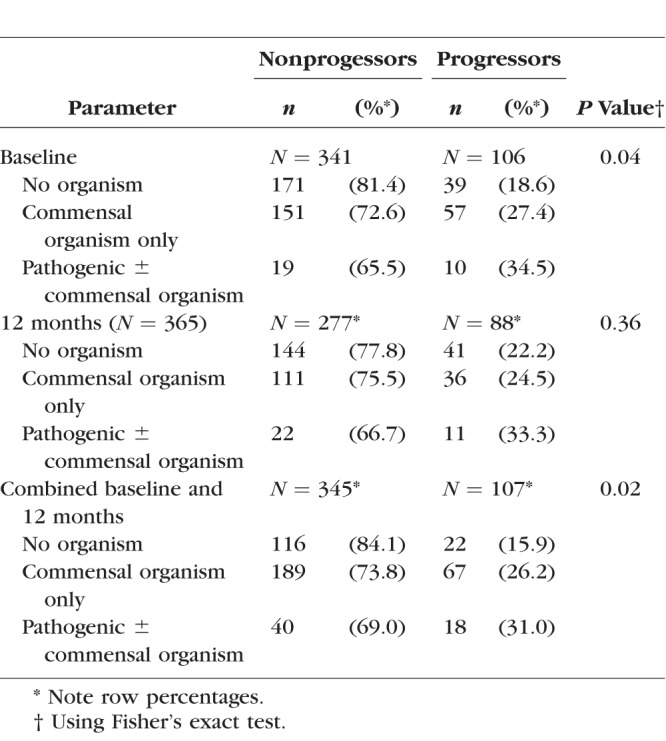
Culture Results at Baseline, 12 Months, and Combined Baseline/12 Months, According to Scarring Progression

**Table 4:**
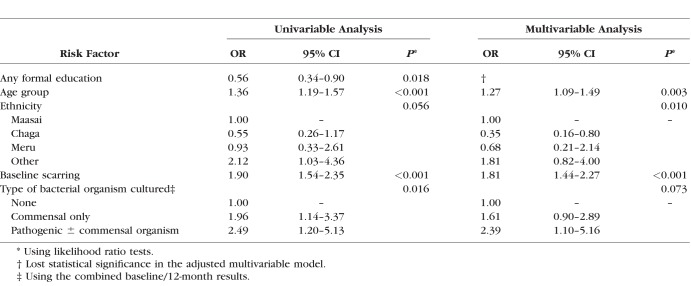
Univariable ORs and Multivariable Logistic Regression Model for Scarring Progression

Table 5 shows progression by combination of pathogenic and commensal organisms. Participants with pathogenic organisms alone were most likely to be progressors (OR = 3.82; 95% CI: 0.98 to 14.84 compared with nonprogressors), although there were only 12 participants in this category.

**Table 5:**
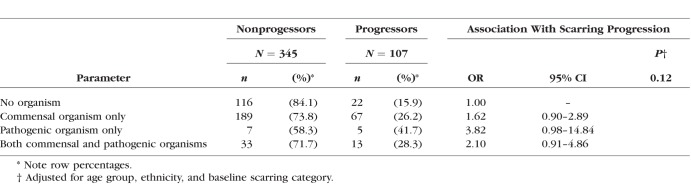
Culture Results Using the Combined Baseline and 12-Month Results According to Scarring Progression: Showing Pathogenic Organisms Only and Pathogenic With Commensal Organisms

## Discussion

There is clear evidence linking conjunctival infection with *C. trachomatis* to active trachoma in children.^2,21^ In contrast, the relationship between *C. trachomatis* infection and the pathogenesis of progressive trachomatous scarring in older people is less clearly defined.^1,7^ In keeping with previous work, in the current study, we only very infrequently detected *C. trachomatis* in adults with scarring. Previous work has shown that non-chlamydial bacterial infection is found more frequently in those with trichiasis and is associated with recurrence of trichiasis after surgery.^3,14–16^ We previously found that individuals with trachomatous conjunctival scarring (without trichiasis) more frequently have conjunctival commensal and pathogenic organisms detected compared with controls.^13^ A recent study found no association between conjunctival scarring and non-chlamydial bacterial carriage.^22^ However, this study was undermined by only 14% of all swabs detecting a bacterial isolate, a much lower detection rate compared with other conjunctival bacteriology studies and by failing to find any association between bacterial carriage and the presence of trichiasis, also in marked contrast to other studies.^3,14–16,23–26^

The study reported in the current paper found some evidence that the presence of non-chlamydial bacteria on the conjunctival surface at baseline/12 months was associated with scarring progression and that the estimated effect of pathogenic organisms was greater than that of commensal organisms. Post hoc analysis found no evidence of a difference between individuals with pathogenic organisms and those with commensal organisms only, and if progression of scarring was modeled using culture of any organism (either commensal or pathogenic), compared with no culture, then an OR for progression (after adjusting for the age group, ethnicity, and baseline scarring) was found of 1.75 (95% CI: 0.99 to 3.06; *P* = 0.05). Differentiating between commensal and pathogenic organisms can be challenging, however. Previous work found that organisms classified as pathogenic were strongly associated with a marked clinical inflammatory response, whereas those classified as commensal were not.^13^ However, organisms normally considered to be commensal at a particular site may, under some conditions, act in pathogenic manner.^27^ It has been postulated that when such bacteria reach a threshold population level, pathogenic mechanisms are triggered.^28^ It is plausible that “commensal” organisms act in a symbiotic manner in the normal, healthy ocular surface, but in eyes with trachomatous conjunctival scarring, they interact with the ocular surface in different ways, leading to proinflammatory effects. For example, previously we found that in eyes with conjunctival scarring, there are marked changes in the expression of several mucins.^11^ This may lead to altered barrier protection by the mucin layer, resulting in more direct interaction between ocular surface bacteria and the epithelium, potentially promoting inflammation.

Bacterial infection of the ocular surface triggers innate immune responses.^29,30^ Previous work has suggested a role for innate immune mechanisms in driving the scarring process in trachoma. Human conjunctival transcriptome studies in both active and scarring trachoma have shown prominent innate immune responses.^11,12^ Animal studies have shown neutrophil infiltration of the genital tract tissue following *C. trachomatis* inoculation and that the intensity of the infiltrate was related to subsequent fibrotic sequelae.^31,32^ Toll-like receptor-2 knockout mice were able to clear infection but had markedly reduced late oviduct pathology.^33^ A guinea pig model of trachoma looking at neutrophil depletion showed less inflammation clinically and fewer mucosal erosions histologically.^34^

Active trachoma is characterized by histologic and molecular inflammatory changes in the conjunctiva, which may be maintained over prolonged periods by repeated infection with *C. trachomatis*.^10,35^ It is possible that with the development of conjunctival scarring, abnormal innate proinflammatory responses also develop.^6^ The anatomical and morphologic changes to the conjunctival surface may render it more susceptible to interaction with bacteria, leading to more inflammation. Conjunctival fibroblasts from patients with scarring trachoma display profibrotic and proinflammatory features, in particular, an increased interleukin-6 expression and secretion.^35^ It is possible that these pathways are stimulated by non-chlamydial bacteria, leading to chronic inflammation and progressive fibrosis.

This study has several limitations. There was some loss to follow-up; however, this is to be expected in a study of this kind, and there were no systematic differences between those followed up and those lost to follow-up. Detection of organisms was limited to those known and looked for, and we did not include a specific culture media for fungi, potentially underestimating their role. The use of molecular detection techniques may potentially have led to different results. The severity of scarring in the study participants was relatively mild, reflecting the mesoendemic level of trachoma in this population. It would be interesting to compare results in a hyperendemic area with more severe conjunctival scarring, as this may reveal a stronger relationship with non-chlamydial bacterial infection. Pathogenic organisms were detected relatively infrequently, and we believe that a larger sample size may well have found a stronger association between scarring progression and infection.

In conclusion, these cohort study data are consistent with the possibility that non-chlamydial bacterial infection is associated with progression of trachomatous conjunctival scarring. We hypothesise that this is through impaired ocular surface defences and increased vulnerability to pro-inflammatory stimulation, although future studies would be needed to formally test this hypothesis.
